# ARE CDX2, BETA-CATENIN AND WNT IMMUNOMARCHERS USEFUL FOR EVALUATING THE CHANCE OF DISEASE PROGRESSION OR EVOLUTION TO DEATH IN PATIENTS WITH COLORECTAL CANCER?

**DOI:** 10.1590/0102-672020200003e1534

**Published:** 2020-12-18

**Authors:** Fabiola Pabst BREMER, Nicolau Gregori CZECZKO, Luiz Martins COLLAÇO, Letícia Elizabeth Augustin Czeczko RUTZ, Guilherme GIONEDIS, Camila Kienen YAMAKAWA

**Affiliations:** 1 Mackenzie Evangelical Faculty of Paraná, Curitiba, PR, Brazil; 2 University Evangelical Mackenzie Hospital, Curitiba, PR, Brazil

**Keywords:** CDX2, Beta-catenin, Wnt3, Colorectal cancer, CDX2, Beta-catenina, Wnt3, Câncer colorretal

## Abstract

**Background:**

Colorectal cancer (CRC) is one of the most common types of cancer in the world. Over time, intestinal epithelial cells undergo mutations that may lead to proliferative advantage and the emergence of cancer. Mutations in the beta-catenin pathway are amongst those described in the development of CRC.

**Aim:**

To verify the existence of a relation between the presence of Wnt3, beta-catenin and CDX2 in colorectal cancer samples and clinical outcomes such as disease progression or death.

**Method:**

Wnt3a, beta-catenin and CDX2 immunohistochemistry was performed on CRC tissue microarray samples (n=122), and analysis regarding the relation between biomarker expression and disease progression or death was performed.

**Results:**

No significant difference was found between the presence or absence of CDX2, beta-catenin or Wnt3a expression and clinical stage, tumor grade, disease progression or death.

**Conclusion:**

CDX2, beta-catenin and Wnt3a are not useful to predict prognosis in patients with CRC.

## Graphical Abstract

[Fig f1a]

**Figure f1a:**
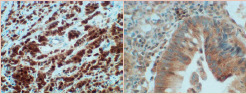
Immunomarking: intra-nuclear beta-catenin and Wnt3a

## RESUMO

**Racional:**

O câncer colorretal (CCR) é um dos tipos mais comuns no mundo. As células epiteliais intestinais podem sofrer mutações que ocasionam vantagem proliferativa e culminam com o surgimento do câncer. Mutações da via da beta-catenina foram descritas entre as que podem ocasioná-lo.

**Objetivo:**

Verificar a existência de relação entre a expressão de Wnt3, beta-catenina e CDX2 em amostras de câncer colorretal com os eventos clínicos progressão de doença e óbito.

**Método:**

Foi realizada análise imunoistoquímica de Wnt3a, beta-catenina e CDX2 em blocos multiamostrais de CRC (n=122), e avaliada a relação entre a expressão dos biomarcadores e os desfechos progressão de doença e óbito.

**Resultados:**

Não foram encontradas diferenças significativas entre a expressão ou ausência de CDX2, beta-catenina ou Wnt3a e estádio clínico, grau de diferenciação tumoral, presença de progressão de doença ou evolução ao óbito.

**Conclusão:**

Os marcadores CDX2, beta-catenina e Wnt3a não são úteis para predizer prognóstico em pacientes com CCR.

## Graphical Abstract

[Fig f1as]

**Figure f1as:**
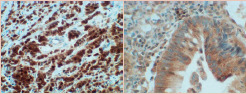
Imunomarcação: beta-catenina intra-nuclear e Wnt3a

## INTRODUCTION

Colorectal cancer (CRC) is the third most common type of cancer in the world, being the third most common in men in Brazil and the second in women^13^. Annually, more than one million diagnoses are performed worldwide, with an estimated mortality of 600,000 individuals per year^23^. In Brazil alone, it is estimated that there will be 20,520 new cases in men and 20,470 in women for each year of the 2020-2022 period. Five-year survival is directly related to staging^9^, and varies from 90%, if localized disease, to 14% in the presence of metastasis^12^ ^,^ ^22^.

The development of CRC begins with the occurrence of mutations in the cells of the intestinal epithelium, which cause proliferative advantages^10^. The increased proliferation leads to the formation of benign adenomatous polyps, which can progress to the genesis of malignant tumors^10^. The time between adenoma and cancer development is about 10 years^5^. Several mutations have already been related to the development of colorectal cancer, including those of the APC genes (adenomatous polyposis coli), KRAS, p53, of the beta-catenin gene, among others^5^.

Wnt proteins are glycoproteins that control cell development, proliferation and death from activation of the Wnt/beta-catenin pathway^25^. In this way, the proteins Wnt1, Wnt3a and Wnt7a would stimulate the inactivation of the formation of the beta-catenin destruction complex (formed by casein kinase 1 - CK1, glycogen synthase kinase 3 - GSK3, axin protein and APC protein), causing intracellular accumulation of beta-catenin^21^. This accumulation leads to the activation of target genes of the Wnt/beta-catenin pathway, responsible for controlling cell proliferation. When this pathway is deregulated, either by hyperstimulation by Wnt or by other mutations that lead to an increase in free intracellular beta-catenin, marked cell proliferation is generated, which can originate CRC ^25^.

The CDX2 transcription factor (caudal type homeobox type 2) is part of the set of proteins encoded by the genes of the homeobox group, which are responsible for the formation of factors essential to the initial development of the embryo, as well as standardization and cell identification^7^. After birth, CDX2 expression becomes important for the morphogenesis of the intestinal epithelium, where it remains present throughout life^4^; therefore, it can be used as a specific marker of colorectal tissue in immunohistochemical studies^16^.

It is believed that, in addition to defining the intestinal phenotype in epithelial cells, CDX2 also has a tumor suppression function^6^, since its absence correlates with less histological differentiation and advanced staging in colorectal malignant tumors^3^ ^,^ ^20^. The mechanism of this suppression is not fully understood, but it is believed that the CDX2 transcription factor acts by blocking the Wnt/beta-catenin signaling pathway^12^.

This research aimed to relate the immunostaining of colorectal cancer samples for CDX2, beta-catenin and Wnt3a with the presence of disease progression and evolution to death.

## METHOD

This study was approved by the Research Ethics Committee of Mackenzie Evangelical Faculty of Paraná, Curitiba, PR, Brazil under no. 1,999,670.

Retrospectively, 122 patients with a diagnosis of colorectal adenocarcinoma performed between the years 2010 and 2015 at the University Evangelical Mackenzie Hospital, Curitiba, PR, Brazil, were included. The tissue samples from these patients were separated in the hospital’s pathology laboratory and sent for immunostaining. For immunohistochemical evaluation, multi-sample blocks were made using the Tissue Tek Quick-Array^TM^ handpiece, which uses clamps coupled with diameters of 1-3 mm to extract the registered area.

The multi-sample blocks allowed up to 60 fragments of the tumor tissues to be obtained, being subsequently processed and submitted to the immunoperoxidase technique, performed by the Benchmark Ultra^TM^ instrument. The readings were taken after amplification of the label by the primary antibodies, by two pathologists at different times. The results were classified as positive (in the presence of marking), negative (in the absence of marking) or indeterminate. The following clones were used for marking: CDX-2 clone EPR2764Y, manufactured by Cell Marque; beta-catenin clone 14, manufactured by Ventana; and polyclonal Wnt3a, manufactured by Genetex.

Data collection was carried out between March and July 2018, through the analysis of physical and electronic medical records, pathological anatomy reports, image exams and reports for requesting high-cost procedures (chemotherapy).

### Statistical analysis

The results of the quantitative variables were described by means, standard deviations, median and minimum and maximum values. Categorical variables were described by frequencies and percentages. Fine and Gray models were adjusted for the analysis of factors associated with the time until disease progression (PEVENT), considering death as a competitive risk. After adjustment, the estimated association measure was the subdistribution hazard ratio (SHR). For the survival analysis, Cox regression models were adjusted and the hazard ratio values ​​were estimated. For both models, the Wald test was used to assess the significance of the variables. Values ​​of p<0.05 indicated statistical significance. The data were analyzed using the computer program Stata/SE v.14.1, Stata Corp LP, USA.

## RESULTS

Of the 122 patients, 63 were men (51.6%) and 59 women (48.4%). Most cases were between 50-70 years old at the time of diagnosis (n=69, 56.6%); the most common degree of histological differentiation was moderately differentiated (n=101, 82.8%). Most cases had advanced staging at the time of diagnosis (UICC III and IV, n=74, 60.7%, Table 1).

**TABLE 1:** Epidemiological data (n=122)

	n	%
Age at diagnosis		
<50 anos	22	18
50-70 anos	69	56.5
>70 anos	31	25.4
UICC (clinical stage)		
0, I ou II	48	39.3
III e IV	74	60.7
Degree of differentiation		
Poorly differentiated	10	8.2
Moderately differentiated	101	82.8
Well differentiated	8	6.6
Indeterminate	3	2.5
Total	122	100

CDX2 expression was found in 67.2% (n=82), beta-catenin in 42.6% (n=52) and Wnt3a in 43.4% (n=53) of the cases. The rate of inconclusive results varied between 11.5 and 15.6% (Table 2). No significant difference was found between the presence or absence of CDX2, beta-catenin and Wnt3a markers with age at diagnosis, gender, clinical stage and degree of tumor differentiation.

**TABLE 2:** Results found in immunostaining

Marker		n	%
CDX2	negative	25	20.5
	positive	82	67.2
	inconclusive	15	12.3
Beta-catenin	negative	56	45.9
	positive	52	42.6
	inconclusive	14	11.5
WNT3a	negative	50	41
	positive	53	43.4
	inconclusive	19	15.6
Total		122	100

An analysis was performed to verify which factors could be related to the presence of progression (Table 3). There was no significant difference regarding the presence or absence of labeling for CDX2, beta-catenin and Wnt3a and the occurrence of disease progression. There were also no statistical differences between age at diagnosis, clinical stage at diagnosis and gender with or without the occurrence of disease progression.

**TABLE 3:** Analysis of variables in relation to the presence of disease progression

Variable	Classification	n	% of cases with progression	p*	SHR	CI 95%
Age at diagnosis	<50 (ref)	22	10 (45.5)			
	50 a 70	69	25 (36.2)	0.225	0.64	0.31-1.31
	>70	31	8 (25.8)	0.143	0.49	0.20-1.27
Gender	Fem (ref)	59	16 (27.1)			
	Male	63	27 (42.9)	0.127	1.63	0.87-3.04
Degree of differentiation (excluded “indet”)	Poorly differentiated (0)	10	1 (10.0)			
	Well differentiated (ref) (2)	8	3 (37.5)	0.241	3.46	0.44-27.5
	Moderate (1)	101	39 (38.6)	0.360	2.90	0.30-28.2
UICC	0/I/II	48	15 (31.2)			
	III/IV	74	28 (37.8)	0.085	1.68	0.93-3.05
CDX2	Negative (ref)	25	9 (36.0)			
	Positive	82	28 (34.1)	0.982	0.99	0.47-2.08
	Inconclusive	15	6 (40.0)	0.489	1.46	0.50-4.26
Beta-catenin	Negative (ref)	56	18 (32.1)			
	Positive	52	21 (40.4)	0.610	1.18	0.63-2.20
	Inconclusive	14	4 (28.6)	0.902	1.08	0.34-3.43
Wnt3	Negative (ref)	50	20 (40.0)			
	Positive	53	17 (32.1)	0.094	0.58	0.30-1.10
	Inconclusive	19	6 (31.6)	0.364	0.66	0.25-1.63

SHR=subdistribution hazard ratio; CI 95%: 95% confidence interval

Regarding the evolution to death, no significant difference was found between the presence or absence of the studied markers and the occurrence of such an outcome. A statistically significant difference was found when assessing the degree of tumor differentiation, staging at diagnosis and the presence of disease progression. The death event was more commonly found in poorly differentiated tumors (HR 17.6; 3.5-88.6), in the more advanced stages (HR 2.52; 1.49-4.25) and in patients who presented progression of disease (HR 5.91; 3.37-10.4, Table 4).

**TABLE 4:** Analysis of variables in relation to the death event

Variable	Classification	n	% death	p*	HR	CI 95%
Age at diagnosis	<50 (ref)	22	9 (40.9%)			
	50 a 70	69	42 (60.9%)	0.361	1.40	0.68-2.88
	>70	31	17 (54.8%)	0.099	1.98	0.88-4.45
Genre	Male (ref)	63	32 (50.8%)			
	Fem	59	36 (61.0%)	0.134	1.44	0.89-2.34
Degree of differentiation (excluded “indet”)	Well differentiated (ref) (2)	8	2 (25.0%)			
	Moderate (1)	101	58 57.4%)	0.078	3.55	0.87-14.6
	Poorly differentiated (0)	10	7 (70.0%)	0.001	17.6	3.5-88.6
UICC (grouped)	0/I/II	48	22 (45.8%)			
	III/IV	74	46 (62.2%)	<0.001	2.52	1.49-4.25
Progression event	No	79	41 (51.9)			
	Yes	43	27 (62.8)	<0.001	5.91	3.37-10.4
CDX2	Negative (ref)	25	9 (36.0%)			
	Positive	82	28 (34.1%)	0.373	1.33	0.71-2.51
	Inconclusive	15	6 (40.0%)	0.855	1.10	0.41-2.94
Beta-catenin	Negative (ref)	56	28 (50.0%)			
	Positive	52	35 (67.3%)	0.480	1.20	0.73-1.97
	Inconclusive	14	5 (35.7%)	0.791	0.88	0.34-2.28
Wnt3	Negative (ref)	50	30 (60.0%)			
	Positive	53	30 (56.6%)	0.204	0.72	0.43-1.20
	Inconclusive	19	8 (42.1%)	0.268	0.64	0.29-1.40

* COX regression model and Wald test, p<0.05

## DISCUSSION

In this research, the absence of CDX2 was found in 20.5% of the samples analyzed, similar to that previously described in the literature, where poor expression or absence of CDX2 was found in 5-29% of the CRC^1^ ^,^ ^2^ ^,^ ^19^. Similarly, Kaimaktchiev et al. found this marker overexpressed in 85.7% of their CRC samples and in 97.9% of their colorectal adenomas^16^.

Contrary to the findings of the present study, previous research had linked the absence of CDX2 expression with less histological differentiation and more advanced stage at diagnosis^2^ ^,^ ^3^ ^,^ ^20^, as well as lower disease-free and progression-free survival rates^3^ ^,^ ^8^ ^,^ ^20^. Nolte et al. demonstrated that the lower expression of CDX2 is inversely related to the probability of certain factors, such as female gender, mucinous histology, higher tumor pathological degree, higher pT staging, higher TNM classification, lower disease-free survival (41% vs. 74% in positive CDX2 tumors) and lower overall survival in five years (33% vs. 59%), maintaining significance even in multivariate analyzes that excluded gender, tumor grade and clinical stage^20^. In this study, no relationship was found between the absence of CDX2 and a significant increase in risk for outcomes such as disease progression, death and progression in clinical staging or degree of tumor differentiation.

The expression of Wnt3a in the present study, in 43.4% of the evaluated samples, was noticeably lower than that described by Qi et al., where 172 (88.2%) of 203 CRC samples demonstrated positive expression of Wnt3a, and the cases with strong expression were related to less differentiated tumors, advanced clinical stage, presence of metastasis and with tumor recurrence^22^. A reduction in overall survival was noted according to the expression of Wnt3a, being 76.4 months for patients without expression of this marker and 41.7 months for those who had strong expression in tumor cells. There was no significant difference in the expression of Wnt3a when assessing patient age, gender or tumor size^22^.

The study by Madison et al. found Wnt3a expressed in 89.9% of CRC cases (weak in 44.7% and strong in 45.2%), and a relationship between positivity for Wnt3a with the presence of vasculogenic mimicry. The presence of it was associated with poorly differentiated tumors (56.6% vs. 7.1% in well-differentiated tumors), advanced clinical stage (58.3% in EC IV vs. 0% in EC I) and the presence of metastasis or recurrence (31.2% vs. 10.7% when there is no metastasis or recurrence). The researchers found no significant difference in the expression of Wnt3a in relation to gender and age, as well as in relation to the degree of tumor differentiation, disease progression, evolution to death or clinical staging at diagnosis. Considering that there is no guide for reading the immunostaining, some samples that were considered weakly positive may have been considered negative.

Nuclear beta-catenin expression was observed in 42.6% of the samples, similar to that found by Morikawa et al, where the positivity of this marker was recorded in 323 (47%) of 724 cases of CRC^18^. Since mutations in the Wnt/beta-catenin pathway are often associated with the development of cancer - but beta-catenin expression is only found in less than half of the tumors - it is thought that there are other mechanisms that demonstrate the activation of this gene pathway^25^.

The present study demonstrated important differences in relation to the recent literature, mainly in relation to the prognosis, since no marker was found among those studied that can be used as an indication of probability for disease progression or evolution to death. This can be explained by possible differences between studies in the interpretation of the samples, since there are no single criteria that standardize the reading of the expression of biomarkers in immunoanalysis.

## CONCLUSION

The tumor markers CDX2, beta-catenin and Wnt3a were not good instruments to assess the chance of disease progression or the possibility of evolution to death in the context of colorectal cancer.
